# *Klebsiella pneumoniae* Volatile Organic Compounds (VOCs) Protect *Artemia salina* from Fish Pathogen *Aeromonas* sp.: A Combined In Vitro, In Vivo, and In Silico Approach

**DOI:** 10.3390/microorganisms11010172

**Published:** 2023-01-10

**Authors:** Md. Liton Mahmud, Shirmin Islam, Suvro Biswas, Md. Golam Mortuza, Gobindo Kumar Paul, Md. Salah Uddin, Md. Akhtar-E-Ekram, Md. Abu Saleh, Shahriar Zaman, Asad Syed, Abdallah M. Elgorban, Nouf S. S. Zaghloul

**Affiliations:** 1 Microbiology Laboratory, Department of Genetic Engineering and Biotechnology, University of Rajshahi, Rajshahi 6205, Bangladesh; 2 Department of Science and Humanities, Bangladesh Army International University of Science and Technology, Cumilla 3500, Bangladesh; 3 Bangladesh Reference Institute for Chemical Measurements (BRICM), Bangladesh Council of Scientific and Industrial Research (BCSIR), Dhaka 1205, Bangladesh; 4 Department of Botany and Microbiology, College of Science, King Saud University, P.O. Box 2455, Riyadh 11451, Saudi Arabia; 5 Bristol Centre for Functional Nanomaterials, HH Wills Physics Laboratory, Tyndall Avenue, University of Bristol, Bristol BS8 1FD, UK

**Keywords:** *Klebsiella pneumoniae*, *Aeromonas* sp., *Artemia salina*, pathogenic infection, antibiotics, protection

## Abstract

Antibiotic resistance is an alarming threat all over the world, and the biofilm formation efficacy of bacteria is making the situation worse. The antagonistic efficacy of *Klebsiella pneumoniae* against one of the known fish pathogens, *Aeromonas* sp., is examined in this study. Moreover, *Aeromonas* sp.’s biofilm formation ability and in vivo pathogenicity on *Artemia salina* are also justified here. Firstly, six selected bacterial strains were used to obtain antimicrobial compounds against this pathogenic strain. Among those, *Klebsiella pneumoniae*, another pathogenic bacterium, surprisingly demonstrated remarkable antagonistic activity against *Aeromonas* sp. in both in vitro and in vivo assays. The biofilm distrusting potentiality of *Klebsiella pneumoniae*’s cell-free supernatants (CFSs) was likewise found to be around 56%. Furthermore, the volatile compounds of *Klebsiella pneumoniae* were identified by GC-MS in order to explore compounds with antibacterial efficacy against *Aeromonas* sp. through an in silico study, where 5′-methylthioadenosine/S-adenosylhomocysteine nucleosidase (MTAN) (PDB: 5B7P) was chosen as a target protein for its unique characteristics and pathogenicity. Several volatile compounds, such as oxime- methoxy-phenyl-, fluoren-9-ol, 3,6-dimethoxy-9-(2-phenylethynyl)-, and 2H-indol-2-one, 1,3-dihydro- showed a strong binding affinity, with free energy of −6.7, −7.1, and −6.4 Kcal/mol, respectively, in complexes with the protein MTAN. Moreover, the root-mean-square deviation, solvent-accessible surface area, radius of gyration, root-mean-square fluctuations, and hydrogen bonds were used to ensure the binding stability of the docked complexes in the atomistic simulation. Thus, *Klebsiella pneumoniae* and its potential compounds can be employed as an alternative to antibiotics for aquaculture, demonstrating their effectiveness in suppressing *Aeromonas* sp.

## 1. Introduction

In nature, fish are sensitive and become stressed rapidly. Fish disease is caused by interactions between the fish, pathogens (viruses, bacteria, and fungi), and environmental factors [1]. Fish disease at the hatchery and rearing stages is a significant stumbling block that must be recognized. Necrotizing fasciitis (NF), ulcers, septicemia, and diarrhea are the most common dangers of bacteria, which ultimately lead to the aquaculture industry facing financial losses. Furthermore, this opportunistic pathogen is frequently associated with human diarrhea. *Aeromonas* septicemia is a bacterial disease that generally occurs in freshwater fish species in tropical waters by an anaerobic, Gram-negative, facultative bacterium called *Aeromonas hydrophila.* Septicemia, infections, and intra-abdominal or respiratory infections are the other main common symptoms in amphibians and humans [2,3,4]. *A. hydrophila* pathogenicity is multifactorial because the disease is caused by a variety of virulence factors, such as biofilm, various types of secretion systems (II, III, and VI) [2], cytotoxins, adhesions, hemolysins, proteases, lipases, etc. [5,6,7].

In the marine environment, antibiotic resistance (AR) is becoming an alarming threat. Many different parts of the world have reported multiple antibiotic resistance (MAR) among *Aeromonas hydrophila* strains [8,9]. A 5′-methylthioadenosine/S-adenosylhomocysteine nucleoside with a DNA methylation ability is a unique protein of *Aeromonas hydrophila* with distinct properties [10]. Hence, this is being studied as a potential target protein for developing new antibiotics. Due to this fact, several alternative sources, such as plant extracts and the antagonistic potentiality of the microbes, etc., are being researched to cope with this hindrance [11,12]. All species produce volatile organic compounds (VOCs), including bacteria, fungi, and plants [13]. These VOCs are small molecules produced by primary and secondary metabolic processes. Several bacterial volatile nitrogenous compounds formed during secondary metabolism have been identified and characterized for their antioxidant, bactericidal, fungicidal, algicidal, and anticancer activities [14,15]. Moreover, microorganisms’ ability to create a wide range of structurally varied volatile chemicals has been known for decades, but their biological functions and antibacterial properties have only lately received attention. Several investigations have shown that microbial volatiles can operate as both information and antimicrobials in long-distance cross-kingdom communication [16,17]. This makes bacteria’s volatile compounds a very promising source of medicines with antibacterial bioactivity.

*Artemia* is a genus of crustaceans that live in saltwater, commonly conveyed as a type of shrimp. *Artemia salina* larvae (also so-called brine shrimp) are considered a pathogen-disease study model for all life stages of marine crustaceans and early developing life stages of marine fishes because they all have a similar immune system [18,19]. Its body has three main segments: the head, thorax, and abdomen [20]. Many authors have considered *Artemia* as just a prototype organism for researching infection biology [21], host–microbe connections, and the influence of chemotherapy agents on illnesses that affect species of shrimp and crabs, in addition to other crustaceans [22,23].

Bioinformatics tools have been used in the current decade to develop medications for a number of ailments. This expands the field for doing various types of research while also saving time and keeping experiment costs to a minimum [24,25]. Computational and bioinformatics screening methodologies have proven useful in identifying natural bioactive chemicals and determining which physicochemical properties are most relevant to their structure–activity connection [26]. Moreover, through several tools such as Pymol, PyRx, Discover Studio, and YASARA, nowadays, it is becoming possible to identify potential drug targets against several diseases, which not only saves time but also reduces the cost of drug development [27,28]

The goal of the research work was to show the antagonistic efficacy of several bacterial strains on *Aeromonas* sp. as well as the biofilm-disrupting efficacy of the CFSs of the bacteria, as biofilm formation is one of the main causes of antibiotic resistance. Furthermore, we analyze volatile compounds through GC-MS to conduct an in silico study to design drugs against the disease-causing proteins.

## 2. Materials and Methods

### 2.1. Bacterial Strains Selection

*Aeromonas* sp. isolated from infected fish was collected at the Microbiology Laboratory, Department of Genetic Engineering and Biotechnology of the University of Rajshahi.

The six selected strains (three Gram-positive and three Gram-negative) listed in Table S1 were also collected at the Microbiology Laboratory, Department of Genetic Engineering and Biotechnology, University of Rajshahi.

### 2.2. In Vitro Antagonism Test with the Selected Strains

The antibacterial activity of the selected bacterial strains (Table S1) against *Aeromonas* sp. was checked using the agar well diffusion method, following [29] with some modifications. Briefly, Mueller Hinton agar plates were prepared, and the suspension of *Aeromonas* sp., cultured in LB, was spread on the plate; then, 5 mm wells were made in the agar plates and filled with 100 µL of the tested strain culture. After that, the plates were incubated at 37 °C for 24–48 h. The appearance of a clear zone indicates the presence of antagonistic activity. The clear zone of inhibition was measured in mm.

### 2.3. Biofilm Formation Assay of the Selected Strains

The biofilm formation assays were carried out following the protocol of N.I. Di Marco [30] with some modifications. In short, overnight cultured *Aeromonas* sp. was allowed to grow on wells of a 96-well microtiter plate (Tarsons, India) containing 100 µL of Luria Bertani (LB) liquid medium for twenty-four hours in an incubator at 37 ºC. Fresh liquid culture (200 µL) was also allowed to grow on a microtiter plate as a negative control (oDc). After the incubation period, the plate was removed, rinsed three times with double distilled water, and air-dried, then oven-dried at 37 °C for one hour (60 min). This step was validated by staining the recovered wells with crystal violet (0.1 for 5 min, and further washed (×3) with tap water). The de-staining step was performed in ethanol and the OD of the suspension was measured at 595 nm using a microplate reader [31,32]. The isolates were categorized as biofilm producers or not, following the parameters described by H. Nirwati et al. [33].

### 2.4. Inhibition of Biofilm Formation by CFSs of the Selected Strains

To investigate the influence of CFSs on biofilm formation using the co-incubation method, as described earlier, here, 100 µL of CFSs (centrifuged 10,000× *g* rpm/8 min at 4 °C to obtain cell-free supernatants) was added with the bacterial inoculum instead of liquid media. The disruption percentage was calculated using the following equation:

Disruption Percentage = (OD control − OD sample) × 100/OD control

### 2.5. Scanning Electronic Microscope (SEM)

The previously described protocol was followed, and bacteria were allowed to grow on glass slides (5 × 5 mm) and cultured in an incubator for twenty-four hours. As a control, biofilms generated in the absence of CFSs were employed. The glass slides were removed after the incubation period, rinsed twice with double distilled water, air-dried, then oven-dried for one hour at 37 °C (60 min). The recovered wells were stained with crystal violet to confirm this phase (0.1%). The glass slide was cleaned twice with double distilled water after sixty minutes.

The slides were air-dried and coated with platinum vapor (thickness of 30 nm) before being examined with a scanning electron microscope (SEM). CFSs were also tested for their ability to destroy pre-formed biofilms. Biofilms were created on pre-sterilized microscopic glass slides, which were then treated with CFSs and cultured for a further twenty-four hours. The slides were fixed and examined under the SEM after the incubation time, as indicated before. All the microscopic observations were made in triplicates.

### 2.6. In Vivo Effect of Bacteria on Brine Shrimp

In vivo pathogenic action of the six selected bacterial strains (*Staphylococcus aureus*, *Lactobacillus* sp. *Bacillus thuringiensis*, *Klebsiella pneumoniae*, *Pseudomonas* sp., *Escherichia coli*) in *Artemia salina* cyst culture was performed. *Artemia* nauplii were axenically hatched, transferred to six-well culture plates, and kept as described above. Immediately after the transfer of the nauplii, the culture water was inoculated one by one with one of the six other bacterial strains at a calculated concentration of 5.4 × 10^6^ CFU/mL.

### 2.7. In Vivo Antagonism Test against Aeromonas *sp.*

An in vivo antagonism test was performed to examine the antagonism action of the six selected bacterial strains against most pathogenic *Aeromonas* sp. in *Artemia salina* cyst culture. *Artemia* nauplii were axenically hatched, transferred to the culture six-well plates, and maintained as described above. Immediately after the transfer of the nauplii, the culture was inoculated with *Aeromas* sp. and one of the six other bacterial strains, one by one, at a calculated concentration of 5.4 × 10^6^ CFU/mL.

### 2.8. Volatile Compounds Analysis through GC-MS

The optimal SPME experimental parameters were determined based on prior investigations with some modifications [34]. Briefly, 1.5 g of NaCl and around 40 mL of bacterial culture were mixed at 80 rpm in a 15 mL vial that was tightly sealed with a PTFE-silicon septum. The fiber was then removed and put right away in the GC for desorption and analysis. Agilent 7890 gas chromatography apparatus and an Agilent 5977 mass selective detector were used for the GC-MS analysis. The volatile compounds were determined using authentic standards, retention indices (rIs), and the NIST 14.0 library. By injecting a homologous sequence of straight-chain alkanes (C6–C30) into a sample, compound retention indices (rIs) were measured (Figure S1).

### 2.9. Ligand and Protein Preparation and Optimization

A total of 77 volatile compounds of *Klebsiella pneumoniae* were identified through GC-MS analysis, and the 3D structures were retrieved from the PubChem database. The energy of all compounds was minimized and converted into the pdbqt format by Open Babel in PyRx version 0.8. [35]. By employing vibrational frequency, a technique known as DFT functional was used to optimize the molecular structure.

The crystal structures of nucleosidase (MTAN) enzymes of *Aeromonas hydrophila* (5b7n) (Resolution: 1.4 Å) were retrieved from the RCSB Protein Data Bank (http://www.rcsb.org) (accessed on 14 July 2022) [36] using the protein preparation wizard of Pymol version 1.1.0 to prepare the modeled protein for docking analysis by removing the water molecules. The prepared file was then converted into the pdbqt format using Open Babel [37].

### 2.10. Molecular Docking Analysis

Auto Dock Vina was used to perform all docking calculations on the nucleosidase (MTAN) enzymes of *Aeromonas hydrophila*, with a ligand selecting a grid box with a radius of 10.0 around the active site region. The grid center points were set to X = 42.21, Y = −34.17, Z = 5.87 and dimension (Å) to X = 45.37, Y = 39.35, Z = 66.30. The grid box dimensions were selected and set up to wrap the protein’s substrate-binding region. Auto Dock Vina’s performance was visualized using the DS visualizer program [38,39].

### 2.11. Pharmacokinetics Properties Analysis

Swiss ADME, AdmetSR, and pkCSM software were used for the prediction of the volatile compounds’ pharmacokinetics [40,41]. The main focus of this section was absorption, distribution, metabolism, excretion, solubility, toxicity, carcinogenicity, and bioavailability, as well as the drug-likeliness property [42].

### 2.12. Molecular Dynamics Simulation

Molecular dynamics simulations of the ligand–protein complexes were conducted using the YASARA dynamics software package and the AMBER14 force field [43,44]. Preliminary cleaning, optimization, and hydrogen bond networking were carried out on the docked complexes. The TIP3P solvation model with periodic boundary conditions was used to generate a cubic simulation cell [45]. The simulation cell’s physiological conditions were set to 298 K, pH 7.4, and a NaCl concentration of 0.9%. The initial minimization of energy was done with a simulated annealing method using the steepest gradient approaches (5000 cycles); 1.25 fs was set as the simulation time step [46]. The particle mesh Ewald (PME) method was used to estimate the long-range electrostatic interactions, with a cutoff radius of 8 Å [47,48,49]. After every 100 ps, the simulation trajectory data were saved. With constant temperature, pressure, and a Berendsen thermostat, the simulations were conducted for 100 ns. To calculate the root-mean-square deviation, root-mean-square fluctuations, solvent-accessible surface area, radius of gyration, and hydrogen bonds, simulation trajectory analyses were conducted [27,50,51,52,53,54,55,56].

### 2.13. Statistical Analysis

After the data were extracted, they were revised, coded, and fed to statistical software IBM SPSS version 22 (SPSS, Inc. Chicago, IL, USA). All statistical analysis was done using two-tailed tests. A *p*-value less than 0.05 was statistically significant.

## 3. Results

### 3.1. In Vitro Antagonism Test of the Selected Bacterial Strains

In vitro antagonism studies using the well diffusion method produced a positive result, with an inhibitory zone visible encircling the macro-colonies (Table 1). *Klebsiella pneumoniae* had moderate/average (17 ± 1) results against *Aeromonas* sp., indicating that the bacteria can produce antibiotics. Moreover, *Bacillus thuringiensis* showed weak inhibition efficacy, and the other four showed no inhibition potentiality.

### 3.2. Capability of Biofilm Formation by the Selected Bacterial Strain

The biofilm formation ability of *Artemia* sp. is shown in Table 2. From the table, it can be seen that *Artemia* sp. has a strong biofilm-forming ability.

### 3.3. Biofilm Inhibition Assay

**Figure 1:**
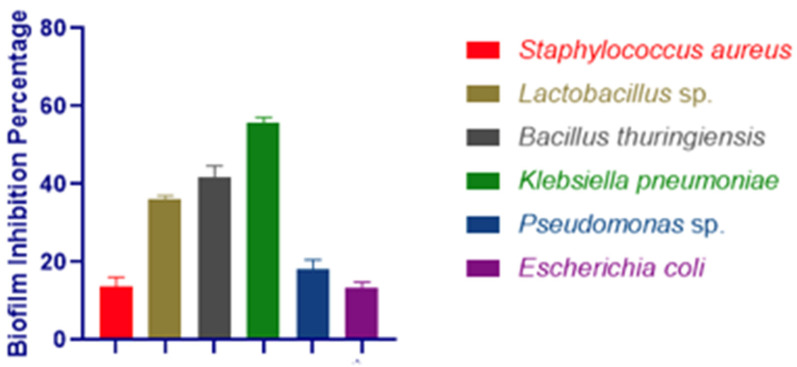
Biofilm inhibition by the CFS from *Staphylococcus aureus*, *Lactobacillus* sp., *Bacillus thuringiensis*, *Klebsiella pneumoniae*, *Pseudomonas* sp. and *Escherichia coli*.

**Figure 2:**
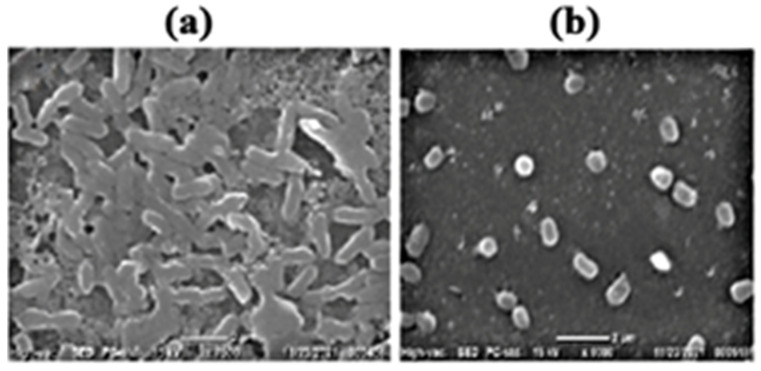
Representative scanning electron micrographs showing biofilm disruption on glass surfaces: (**a**) biofilm formed by *Aeromonas* sp.; (**b**) biofilm disruption by co-incubation with 100 µL of CFS form *Klebsiella pneumoniae* and bacterial culture of *Aeromonas* sp.

*Artemia* sp. has a high propensity to form biofilms. The biofilm inhibition efficacy of the selected six strains showed that only *Klebsiella pneumoniae* had above 50% pre-formed biofilm inhibition efficacy (Figure 1). Furthermore, the production of biofilms was validated using a scanning electron microscope (SEM) (Figure 2).

### 3.4. Effect of Selected Bacterial Strains on Artemia salina

**Figure 3:**
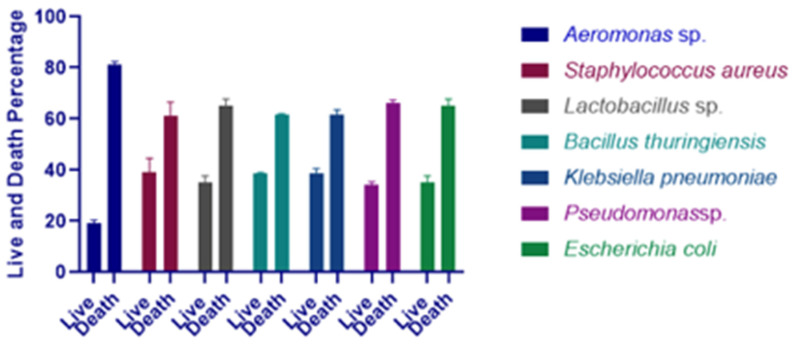
Percentage survival of *A. salina* larvae after 24 h of infection with *Aeromonas* sp., *Staphylococcus aureus*, *Lactobacillus* sp., *Bacillus thuringiensis*, *Klebsiella pneumoniae*, *Pseudomonas* sp., and *Escherichia coli*.

After an experimental infection with bacterial strains *Aeromonas* sp., *Staphylococcus aureus, Lactobacillus* sp. *Bacillus thuringiensis, Klebsiella pneumoniae, Pseudomonas* sp., *Escherichia coli* at a concentration of 5.4 × 10^6^ CFU/mL, all of the *Artemia* larvae (ten cysts of *Artemia salina* used for treatment) died within forty-eight hours of infection. No *Artemia salina* perished after six hours of infection with pathogenic bacteria (concentration 5.4 × 10^6^ CFU/mL). After twenty-four hours of infection with those bacteria, the mortality rate was 81%, 61, 65%, 61.5%, 61.5%, and 66%, respectively (Figure 3). Hence, *Aeromonas* sp. was chosen as the most pathogenic bacterium for *Artemia* in the next study.

### 3.5. Effect of Single and Dual Bacteria on Artemia salina

**Figure 4:**
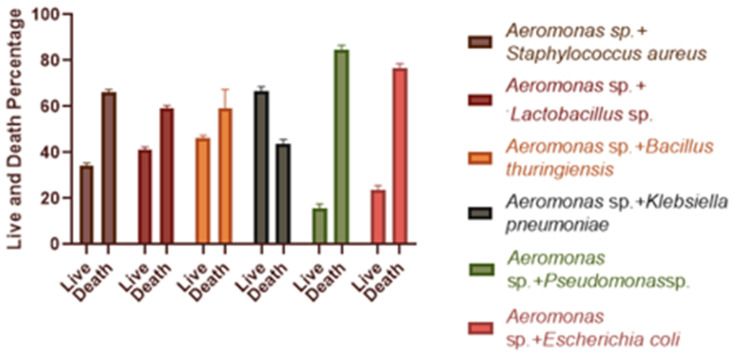
Percentage survival of *A. salina* larvae infected with two strains at a time. Here, *Aeromonas* sp. was used as a constant pathogen, and the other six were added to decipher the antagonistic effect. Data were recorded after 24 h of infection. Error bars represent standard deviation.

After 24 h of infection, *Artemia salina* was found to be alive. Figure 4 depicts the findings of the in vivo antagonism test, displaying cultures injected with the specified strains as well as *Aeromonas* sp. at a concentration of 5.4 × 10^6^ CFU/mL. When compared to the solo *Aeromonas* sp. treatment, the survival rate for each combination was higher. The largest percentage of *Artemia salina* survived when *Klebsiella pneumoniae* and *Aeromonas* sp. were combined (around 66.5 %).

### 3.6. Determination of Binding Energy

We performed a molecular docking analysis of a total of 77 volatile compounds produced by *Klebsiella pneumoniae* (docking against the nucleosidase (MTAN) enzymes of *Aeromonas hydrophila* (5b7n)) (Table S2). The highest docking score was −7.1 kcal/mol (L-32), and the lowest score was −2.3 kcal/mol (L-15). Interestingly, 7 volatile compounds out of 77 had a docking score of −7.1 to −6.0 kcal/mol (Table 3).

### 3.7. Protein–Ligand Interaction

After molecular docking, binding interactions between the active sites of ligands and proteins were considered. Table 4 and Table S3 indicate that the protein and ligands were linked by two types of bonds, such as H bonding interactions and hydrophobic bonds. The basic reason for the strong hydrogen bond effect is due to its shorter bond distance (~3.0 Å), which is less than other bond distances. On the other hand, some types of hydrophobic bonds that have been formed are considered to be weak bonds because their bond distance is between 4.0 and 5.0 Å. For each protein–ligand interaction, it was found that the number of hydrophobic bonds was two to three times greater than the number of hydrogen bonds, resulting in a binding affinity value.

### 3.8. Pharmacological Properties Assessment

From the ADMET study, the pharmacokinetics properties (Molecular weight, Num. H-bond acceptors, Num. H-bond donors, lop P, log S, TPSA BBB permeability, Human Intestinal Absorption) of the top eight compounds are displayed in Table 5. The results depict that all of the compounds’ molecular weights were less than 500Da. To be considered a potential drug candidate, the topological polar surface area (TPSA) score must be between 0 and 140 [57]. In those compounds, the TPSA values were 0 to 52.82.

### 3.9. Confirmation of the Stability of Ligand–Protein Complexes

**Figure 5:**
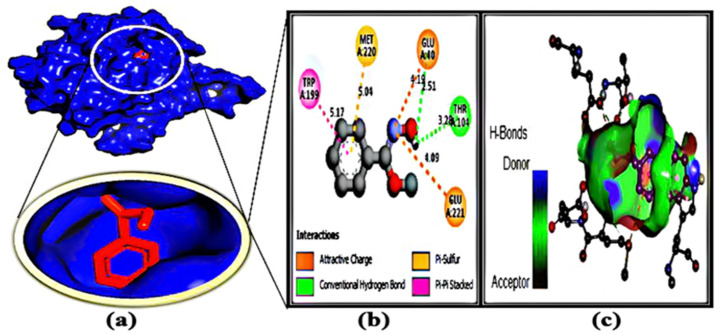
Docking simulation between protein–ligand interactions of nucleosidase (MTAN) enzymes and oxime-, methoxy-phenyl-, respectively, with (**a**) cartoon view (binding pocket), (**b**) 2D view, and (**c**) hydrogen bonding.

**Figure 6:**
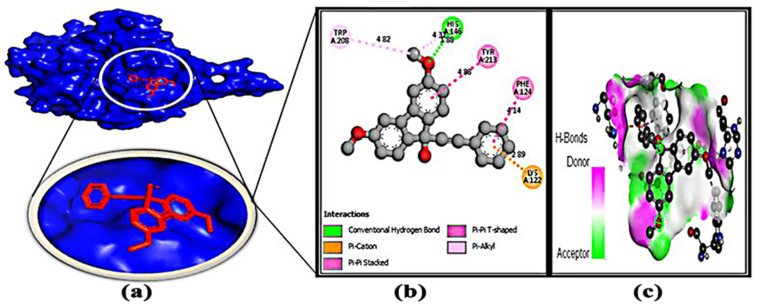
Docking simulation between protein–ligand interactions of nucleosidase (MTAN) enzymes and fluoren-9-ol, 3,6-dimethoxy-9-(2-phenylethynyl)-, respectively, with (**a**) cartoon view (binding pocket), (**b**) 2D view, and (**c**) hydrogen bonding.

**Figure 7:**
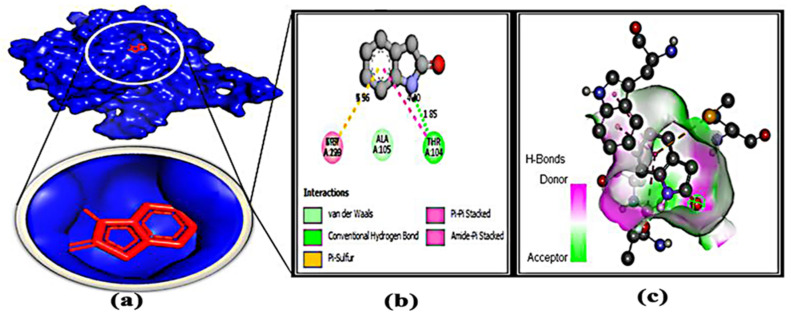
Docking simulation between protein–ligand interactions of nucleosidase (MTAN) enzymes and 2H-indol-2-one, 1,3-dihydro-, respectively, with (**a**) cartoon view (binding pocket), (**b**) 2D view, and (**c**) hydrogen bonding.

**Figure 8:**
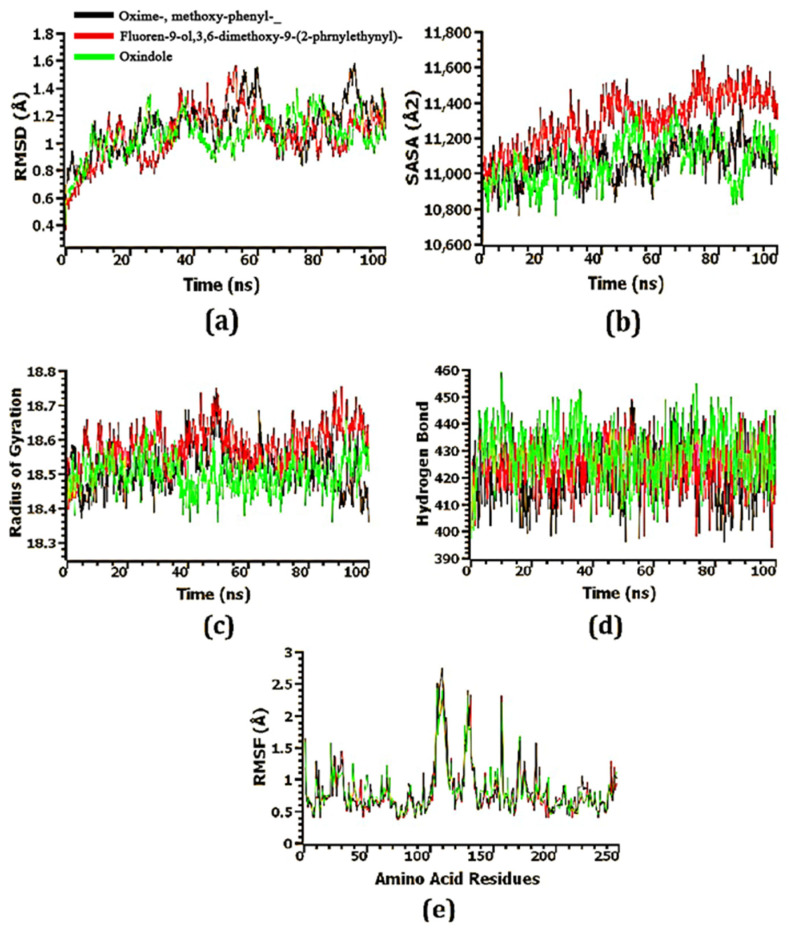
The molecular dynamics simulation of the top 3 docked complexes. (**a**) RMSD, (**b**) SASA, (**c**) radius of gyration, (**d**) hydrogen bond, and (**e**) RMSF.

The structural stability of the docked ligand–protein complexes was investigated using molecular dynamics simulations. To explain variations in the protein–ligand complex’s stability, the RMSD of the C-alpha atoms from simulated trajectories was calculated. According to Figure 5, Figure 6 and Figure 7, the ligand–protein complexes involving oxime-, methoxy-phenyl-, fluoren-9-ol, 3,6-dimethoxy-9-(2-phenylethynyl)-, and oxindole showed an initial upward RMSD trend, which suggests the ligand–protein complexes were flexible. The oxindole-MTA/SAH nucleosidase complex’s RMSD profile dropped dramatically after around 40 ns, then steadied at 50 ns, and remained stable for the last 50 ns of the simulations, with only minor fluctuations. At 35–55 ns, the fluoren-9-ol, 3,6-dimethoxy-9-(2-phenylethynyl)-MTA/SAH nucleosidase complex had a higher RMSD value than the other two complexes, which might explain the complexes’ increased flexibility. In addition to the RMSD at 80–100 ns, the oxime-, methoxy-phenyl-_-MTA/SAH nucleosidase complex had the highest RMSD value, explaining its higher flexibility as well. Nevertheless, the RMSD value of all three ligand–protein complexes was less than 2.5 Å, meaning that the complexes were stable throughout the simulation period (Figure 8a).

To further understand the variations in the protein’s surface area, the solvent-accessible surface area (SASA) of the complexes was also studied. As the SASA increases, the surface area expands, while as it decreases, the protein is truncated. The SASA of the fluoren-9-ol, 3,6-dimethoxy-9-(2-phenylethynyl)-MTA/SAH nucleosidase complex was greater than that of the other two complexes, indicating that the surface area of the complexes was extended (Figure 8b). After 80 ns, the SASA of the oxindole-MTA/SAH nucleosidase complex dropped dramatically before rising again at 95 ns. The oxime-, methoxy-phenyl-_-MTA/SAH nucleosidase complex and the fluoren-9-ol, 3,6-dimethoxy-9-(2-phenylethynyl)-MTA/SAH nucleosidase complex achieved steady-state after 65 and 75 ns, respectively, and remained stable throughout the simulation duration.

The labile nature of the top three complexes was determined using the Rg profile. Due to the folding or unfolding process of the protein, a greater Rg profile corresponds to more flexibility. A lower Rg profile indicates that the simulated complexes are less labile and have a higher stiffness. Despite stable Rg characteristics for the three complexes, the oxime-, methoxy-phenyl-_-MTA/SAH nucleosidase complex showed a decrease in Rg during the 90–100 ns time frame (Figure 8c).

Considering that hydrogen bonds are essential to protein integrity and stability, the docked complexes were analyzed for their hydrogen bonds. Throughout the entire simulation trajectory, the oxime-, methoxy-phenyl-_-MTA/SAH nucleosidase complex, fluoren-9-ol, 3,6-dimethoxy-9-(2-phenylethynyl)-MTA/SAH nucleosidase complex, and oxindole-MTA/SAH nucleosidase complex formed a large number of hydrogen bonds, indicating that the ligand molecule bonded tightly to the MTA/SAH nucleosidase protein (Figure 8d).

To understand the flexibility of the complexes, the root-mean-square fluctuations (RMSFs) were examined. Figure 8e shows that the RMSF descriptor profiles of practically every amino acid residue in three complexes were below 3 Å, indicating that the complexes were stable as low RMSF values are linked with a decreased degree of flexibility.

## 4. Discussion

Due to the outbreak of diseases, aquaculture farmers have been suffering from significant economic losses all over the world. The spread of pathogenic fish bacteria and the associated disease outbreaks are serious limitations in the fish farming industry [58]. In this study, we try to use the antagonism effectivity of selected bacterial strains to control the fish pathogen *Aeromonas* sp. through in vitro, in vivo, and in silico assays. Moreover, in previous findings, *Aeromonas* sp. has also been considered to be highly pathogenic for fish [59,60].

Antibiotic resistance is outpacing the discovery and development of new antibiotics, and the incidence of bacterial diseases that are wholly untreatable with currently available antibiotics has highlighted the ramifications of widespread antibiotic abuse. As a result, it is critical to use alternatives to the available antibiotics [61]. Microbial control of those pathogens may be an alternative method of control [62]. Antimicrobials may be used to treat infected fish as well as a preventative precaution, in addition to disinfectants and biocides. For this reason, in vitro and in vivo antagonistic tests were carried out to identify potential candidates, and in both assays, *Klebsiella pneumoniae* was found to have potential antagonistic efficacy. The in vivo test was carried out in brine shrimp nauplii, belonging to crustaceans, as they are considered a model organism due to physiological similarities with other crustaceans and toxicity testing [63,64]. When *Artemia salina* was treated with *Aeromonas* sp. and *Klebsiella pneumoniae,* its survival rate increased to around 50% (Figure 4). Similar in vivo tests were carried out by Faseela Hamza, who employed *Artemia salina* to determine the bacteria’s pathogenicity [65].

Moreover, biofilm formation is one of the main causes of antibiotic resistance in marine pathogens [66] and the CFS of the bacteria has the potential to produce antibiotics [67]. Here, CFSs of the *Klebsiella pneumoniae* showed a 55.66 ± 1.52% biofilm-disrupting ability where *Aeromonas* sp. had formed strong biofilms. From previous studies, it has been proven that bacterial CFSs have biofilm-disrupting efficacy. CFSs of *B. licheniformis* were found to have biofilm-disrupting activity [65]; 73.08 ± 2.41% and 73.76 ± 1.06% biofilm disruption ability of the CFSs of *B. licheniformis* were observed for *V. harveyi* and *P. aeruginosa* by Faseela Hamza et al. [67].

Microorganisms are a source of new antibiotics to address antibiotic resistance problems [68]. Microorganisms’ ability to create a wide range of structurally varied volatile chemicals has been known for decades, but their biological functions and antibacterial actions have just lately come to light. Several investigations have shown that microbial volatiles can operate as both information and antimicrobials in long-distance cross-kingdom communication and competition and predation [17,69]. For deciphering the potential compounds, 77 volatile compounds of *Klebsiella pneumoniae* were identified (Figure S1).

Bioinformatics is becoming a promising tool for identifying potential drug candidates [70]. In order to develop new drugs against aerolysin and stop it from binding to hemolysin proteins, molecular docking has provided useful information on the identification of crucial interacting sites [7]. Hence, the identification of the potential protein of *Aeromonas* sp. could be a potential target to deactivate the protein related to pathogenicity. Here, the methylthioadenosine/s-adenosylhomocysteine nucleosidase (PDB: 5B7P) was chosen as a potential drug target due to its unique properties [10]. Several studies have been done to inactivate several proteins of *Aeromonas* sp. Toja et al. selected ftsZ and ftsY proteins as a target, and phytochemicals of *haplolobus monticola* were selected to inactivate those proteins [1]. Aerolysin was considered a potential target by Sunita Kumari Yadav [7], but no work was done on the methylthioadenosine/s-adenosylhomocysteine nucleosidase to grab attention as a potential drug target. Moreover, the volatile compound 1-(9H-fluoren-2-yl)-2-(1-phenyl-1H-ttetrazole5-ylsulfanyl)-ethanone from *Bacillus subtilis* was identified as a potential anti-biofilm compound to combat antibiotic resistance brought on by *Pseudomonas* sp. through in silico studies [14]. Here, 77 compounds were docked through Auto Dock Vina (Table S2), 8 potential compounds were selected, and 3 of the most competent compounds were selected for simulation. From all the parameters, oxime-, methoxy-phenyl-, fluoren-9-ol, 3,6-dimethoxy-9-(2-phenylethynyl)-, and 2H-Indol-2-one, 1,3-dihydro- were identified as potential drug candidates (Figure 5, Figure 6, Figure 7 and Figure 8). Moreover, their pharmacokinetics properties were examined to obtain the drug-like properties of the compounds; the compounds also had drug-likeliness (Table 3). Natural products from microbes have been gaining the attention of the scientific community recently. In a previous study, the authors found several potential compounds against SARS-CoV-2 from a retrieved microbial natural compounds database through an in silico study [71]. However, still, there are no other works where volatile compounds from bacteria have been identified as potential compounds against any disease. Hence, it can be assumed that *Klebsiella pneumoniae* has antagonistic efficacy against *Aeromonas* sp.; the result was confirmed through in vivo, in vitro, and in silico studies. Moreover, the volatile compounds, named oxime- methoxy-phenyl-_, fluoren-9-ol, 3,6-dimethoxy-9-(2-phenylethynyl)-, and 2H-Indol-2-one, 1,3-dihydro, could be potential compounds for creating new antibiotics.

## 5. Conclusions

The presence of antibacterial compounds in *Klebsiella pneumoniae* has been proved through the in vitro well diffusion method and in vivo *Artemia salina* assays. Moreover, through in silico studies such as docking score, ADMET, and RMSD values, oxime- methoxy-phenyl-, fluoren-9-ol, 3,6-dimethoxy-9-(2-phenylethynyl)-, and 2H-indol-2-one, 3-dihydro have been identified as potential drug candidates against the pathogenic protein of *Aeromonas* sp. However, in vivo validation is needed before approving those compounds as potential drug candidates.

## Figures and Tables

**Table 1:** Antagonistic activity of the six selected strains against *Aeromonas* sp.

Bacterial Name	Zone of Inhibition (mm)	Status
*Staphylococcus aureus*	6	No inhibitory activity (−)
*Lactobacillus* sp.	7.5 ± 1	No inhibitory activity (−)
*Bacillus thuringiensis*	12 ± 1	Weak inhibition (+)
*Klebsiella pneumoniae*	17 ± 1	Moderate/average inhibition (++)
*Pseudomonas* sp.	7.33 ± 0.57	No inhibitory activity (−)
*Escherichia coli*	6	No inhibitory activity (−)

**Table 2:** The biofilm formation efficacy of *Aeromonas* sp.

Name of the Bacteria	Range	Result
*Aeromonas* sp.	OD > 4 × ODcutoff	Strong

**Table 3:** Ligand no. and PubChem CID of the top 8 molecules based on docking score binding affinity.

S/N	Ligand No	PubChem CID	Docking Score (Binding Affinity) Kcal/mol
1	L-32	631096	−7.1
2	L-8	9602988	−6.7
3	L-58	321710	−6.4
4	L-41	139743	−6.3
5	L-37	931	−6.2
6	L-17	548611	−6.1
7	L-31	6054	−6.0
8	L-76	545303	−5.9

**Table 4:** Protein–ligand interactions of nucleosidase (MTAN) enzymes (PDB ID: 5b7n) with the top 8 volatile compounds of *Klebsiella Pneumoniae*.

SI NO	Compound	Hydrogen Bond		Hydrophobic Bond	
		Residues	Distance (A°)	Residues	Distance (A°)
**1.**	L-32	HIS-146	1.88	TRP-208 TYR-213 PHE-124 LYS-122	4.82 4.88 4.14 2.89
**2.**	L-8	THR-104	3.28	MET-220 GLU-40 GLU-221 TRP-199	5.04 4.19 4.09 5.17
**3.**	L-58	THR-104	1.85	MET-220	5.96
**4.**	L-41	SER-243 ASN-244	2.74 2.11	VAL-78 ALA-36 GLU-218 TRP-199 MET-37 MET-27 MET-220	5.21 5.38 3.72 3.75 4.95 4.95
**5.**	L-37	Absent	Absent	TRP-199	4.45
**6.**	L-17	SER-28 VAL-71 GLY-64	2.55 2.20 2.45	GLN-63 PRO-70 PRO-30 ALA-29	2.85 5.28 5.37 3.80
**7.**	L-31	ARG-240	2.50	MET-220 MET-220 TRP-199 ARG-240	1.39 5.05 5.05 2.50
**8.**	L-76	TRP-145	2.11	TRP-145 TRP-145 TRP-145 VAL-140	3.73 3.73 4.60 4.67

**Table 5:** Pharmacological assessment of the screened hit ligand molecules. Here, Ac indicates hydrogen bond acceptor; H. Do indicates hydrogen bond donor; TPSA indicates topological polar surface area; BBB indicates blood–brain barrier.

Ligand	L\N	MW g/mol	H. Ac	H. Do	Log Po/w	Log S	Number of Lipinski violations	TPSA (Å^2^)	Human Intestinal Absorption)	BBB (+ve/−ve)
L-32	1	342.4	3	2	4.26	−5.03	Yes; 0 violation	38.69	High	+ve
L-8	2	151.16	3	1	2.03	−2.33	Yes; 2 violations	41.82	High	+ve
L-58	3	133.15	1	1	1.16	−1.84	Yes; 2 violations	29.10	High	+ve
L-41	4	156.22	2	0	2.79	−2.37	Yes; 2 violations	26.30	High	+ve
L-37	5	128.17	0	0	3.30	−3.45	Yes; 2 violations	0.00	Low	+ve
L-17	6	282.12	3	2	3.77	−4.33	Yes; 0 violation	52.82	High	+ve
L-31	7	122.16	1	1	1.36	−1.82	Yes; 2 violations	20.23	High	+ve
L-76	8	276.37	3	0	3.81	−3.82	Yes; 0 violation	43.37	High	+ve

## Data Availability

All data generated or analyzed during this study are included in this published article and its Supplementary Information Files.

## Supplementary Materials

The following supporting information can be downloaded at: https://www.mdpi.com/article/10.3390/microorganisms11010172/s1, Figure S1: GC-MS chromatogram of volatile compounds produced by *Klebsiella pneumoniae*; Table S1: List of bacterial strains used for antagonistic test; Table S2: Volatile compounds (with PubChem ID, molecular weight and binding affinity) produced by *Klebsiella pneumoniae*. Data were obtained form GC-MS analysis; Table S3: Protein-ligand interaction of top eight hit molecules with their binding residues.

## References

[1] Toja Y.T., Suprayitno E., Yanuhar U. (2020). In Silico Potential Black Fruit Seeds (Haplolobus Monticola) Wondama Local Plant West Papua Barat as Antibacterial Aeromonas Hydrophila. Eurasian J. Biosci..

[2] Moriel B., de Campos Prediger K., de Souza E.M., Pedrosa F.O., Fadel-Picheth C.M.T., Cruz L.M. (2021). In Silico Comparative Analysis of Aeromonas Type VI Secretion System. Braz. J. Microbiol..

[3] Fernández-Bravo A., Figueras M.J. (2020). An Update on the Genus Aeromonas: Taxonomy, Epidemiology, and Pathogenicity. Microorganisms.

[4] Janda J.M., Abbott S.L. (2010). The Genus Aeromonas: Taxonomy, Pathogenicity, and Infection. Clin. Microbiol. Rev..

[5] Teunis P., Figueras M.J. (2016). Reassessment of the Enteropathogenicity of Mesophilic Aeromonas Species. Front. Microbiol..

[6] Venkatasamy V., Durairaj R., Karuppaiah P., Sridhar A., Kamaraj S.K., Ramasamy T. (2021). An In Silico Evaluation of Molecular Interaction Between Antimicrobial Peptide Subtilosin A of Bacillus Subtilis with Virulent Proteins of Aeromonas Hydrophila. Int. J. Pept. Res. Ther..

[7] Yadav S.K., Panwar D., Singh A., Tellis M.B., Joshi R.S., Dixit A. (2021). Molecular Phylogeny, Structure Modeling and in Silico Screening of Putative Inhibitors of Aerolysin of Aeromonas Hydrophila EUS112. J. Biomol. Struct. Dyn..

[8] Vivekanandhan G., Savithamani K., Hatha A.A.M., Lakshmanaperumalsamy P. (2002). Antibiotic Resistance of Aeromonas Hydrophila Isolated from Marketed Fish and Prawn of South India. Int. J. Food Microbiol..

[9] Odeyemi O.A., Asmat A., Usup G. (2012). Antibiotics Resistance and Putative Virulence Factors of Aeromonas Hydrophila Isolated from Estuary. J. Microbiol..

[10] Xu Y., Wang L., Chen J., Zhao J., Fan S., Dong Y., Ha N.C., Quan C. (2017). Structural and Functional Analyses of Periplasmic 5′-Methylthioadenosine/S-Adenosylhomocysteine Nucleosidase from Aeromonas Hydrophila. Biochemistry.

[11] Hassan M., Kjos M., Nes I.F., Diep D.B., Lotfipour F. (2012). Natural Antimicrobial Peptides from Bacteria: Characteristics and Potential Applications to Fight against Antibiotic Resistance. J. Appl. Microbiol..

[12] Nascimento G.G.F., Locatelli J., Freitas P.C., Silva G.L. (2000). Antibacterial Activity of Plant Extracts and Phytochemicals on Antibiotic-Resistant Bacteria. Braz. J. Microbiol..

[13] Ossowicki A., Jafra S., Garbeva P. (2017). The Antimicrobial Volatile Power of the Rhizospheric Isolate Pseudomonas Donghuensis P482. PLoS ONE.

[14] Islam1 S., Mahmud1 M.L., Almalki W.H., Biswas S., Islam M.A., Mortuza M.G., Hossain M.A., Ekram M.A.-E., Uddin M.S., Zaman S. (2022). Cell-Free Supernatants (CFSs) from the Culture of Bacillus Subtilis Inhibit Pseudomonas Sp. Biofilm Formation. Microorganisms.

[15] Valença C.A.S., Barbosa A.A.T., Souto E.B., Caramão E.B., Jain S. (2021). Volatile Nitrogenous Compounds from Bacteria: Source of Novel Bioactive Compounds. Chem. Biodivers..

[16] Lammers A., Lalk M., Garbeva P. (2022). Air Ambulance: Antimicrobial Power of Bacterial Volatiles. Antibiotics.

[17] Avalos M., van Wezel G.P., Raaijmakers J.M., Garbeva P. (2018). Healthy Scents: Microbial Volatiles as New Frontier in Antibiotic Research?. Curr. Opin. Microbiol..

[18] Napolitano G., Motta C.M., Agnisola C., Venditti P., Fasciolo G., Ferrandino I., Capriello T., Vitale E., Costanzo G., Avallone B. (2022). Commercial Red Food Dyes Preparations Modulate the Oxidative State in Three Model Organisms (Cucumis Sativus, Artemia Salina, and Danio Rerio). Environments.

[19] Mohd-Aris A., Muhamad-Sofie M.H.N., Zamri-Saad M., Daud H.M., Yasin Ina-Salwany M. (2019). Live Vaccines against Bacterial Fish Diseases: A Review. Vet. World.

[20] Rodrigues C.M., Bio A.M., Amat F.D., Monteiro N.M., Vieira N.M. (2012). Surviving an Invasion: Characterization of One of the Last Refugia for Artemia Diploid Parthenogenetic Strains. Wetlands.

[21] Overton S.V., Bland C.E. (1981). Infection of Artemia Salina by Haliphthoros Milfordensis: A Scanning and Transmission Electron Microscope Study. J. Invertebr. Pathol..

[22] Morya V.K., Choi W., Kim E.K. (2014). Isolation and Characterization of Pseudoalteromonas Sp from Fermented Korean Food, as an Antagonist to Vibrio Harveyi. Appl. Microbiol. Biotechnol..

[23] Verschuere L., Rombaut G., Sorgeloos P., Verstraete W. (2000). Probiotic Bacteria as Biological Control Agents in Aquaculture. Microbiol. Mol. Biol. Rev..

[24] Guo R., Zhao Y., Zou Q., Fang X., Peng S. (2018). Bioinformatics Applications on Apache Spark. Gigascience.

[25] Dao F.Y., Lv H., Wang F., Feng C.Q., Ding H., Chen W., Lin H. (2019). Identify Origin of Replication in Saccharomyces Cerevisiae Using Two-Step Feature Selection Technique. Bioinformatics.

[26] Ivanov J., Polshakov D., Kato-Weinstein J., Zhou Q., Li Y., Granet R., Garner L., Deng Y., Liu C., Albaiu D. (2020). Quantitative Structure−activity Relationship Machine Learning Models and Their Applications for Identifying Viral 3Clpro- And RDRP-Targeting Compounds as Potential Therapeutics for Covid-19 and Related Viral Infections. ACS Omega.

[27] Mahmud S., Paul G.K., Afroze M., Islam S., Gupt S.B.R., Razu M.H., Biswas S., Zaman S., Uddin M.S., Khan M. (2021). Efficacy of Phytochemicals Derived from Avicennia Officinalis for the Management of Covid-19: A Combined in Silico and Biochemical Study. Molecules.

[28] Scieuzo C., Nardiello M., Farina D., Scala A., Cammack J.A., Tomberlin J.K., Vogel H., Salvia R., Persaud K., Falabella P. (2021). Hermetia Illucens (L.) (Diptera: Stratiomyidae) Odorant Binding Proteins and Their Interactions with Selected Volatile Organic Compounds: An in Silico Approach. Insects.

[29] Karimi S., Rashidian E., Birjandi M., Mahmoodnia L. (2018). Antagonistic Effect of Isolated Probiotic Bacteria from Natural Sources against Intestinal Escherichia Coli Pathotypes. Electron. Physician.

[30] Di Marco N.I., Pungitore C.R., Lucero-Estrada C.S.M. (2020). Aporphinoid Alkaloids Inhibit Biofilm Formation of Yersinia Enterocolitica Isolated from Sausages. J. Appl. Microbiol..

[31] Saito Y., Fujii R., Nakagawa K.I., Kuramitsu H.K., Okuda K., Ishihara K. (2008). Stimulation of Fusobacterium Nucleatum Biofilm Formation by Porphyromonas Gingivalis. Oral Microbiol. Immunol..

[32] O’Toole G.A. (2010). Microtiter Dish Biofilm Formation Assay. J. Vis. Exp..

[33] Nirwati H., Sinanjung K., Fahrunissa F., Wijaya F., Napitupulu S., Hati V.P., Hakim M.S., Meliala A., Aman A.T., Nuryastuti T. (2019). Biofilm Formation and Antibiotic Resistance of Klebsiella Pneumoniae Isolated from Clinical Samples in a Tertiary Care Hospital, Klaten, Indonesia. BMC Proc..

[34] Junfen L.I.N., Mengna W.U., Haocheng W.U., ZHANG T., Chen W.U., Fudong L.I. (2020). Epidemiological Characteristics of Coronavirus Disease 2019 in Zhejiang Province. J. Prev. Med..

[35] Rakib A., Nain Z., Sami S.A., Mahmud S., Islam A., Ahmed S., Siddiqui A.B.F., Babu S.M.O.F., Hossain P., Shahriar A. (2021). A Molecular Modelling Approach for Identifying Antiviral Selenium-Containing Heterocyclic Compounds That Inhibit the Main Protease of SARS-CoV-2: An in Silico Investigation. Brief. Bioinform..

[36] Bueno M.D.L.G.B. (2018). Molecular Docking, Pharmacokinetic, and DFT Calculation of Naproxen and Its Degradants. Biomed. J. Sci. Tech. Res..

[37] Hashem H.E., Nath A., Kumer A. (2021). Synthesis, Molecular Docking, Molecular Dynamic, Quantum Calculation, and Antibacterial Activity of New Schiff Base-Metal Complexes. J. Mol. Struct..

[38] Ghosh R., Chakraborty A., Biswas A., Chowdhuri S. (2020). Depicting the Inhibitory Potential of Polyphenols from Isatis Indigotica Root against the Main Protease of SARS CoV-2 Using Computational Approaches. J. Biomol. Struct. Dyn..

[39] Nath A., Kumer A., Zaben F., Khan M.W. (2021). Investigating the Binding Affinity, Molecular Dynamics, and ADMET Properties of 2,3-Dihydrobenzofuran Derivatives as an Inhibitor of Fungi, Bacteria, and Virus Protein. Beni-Suef Univ. J. Basic Appl. Sci..

[40] Daina A., Michielin O., Zoete V. (2017). SwissADME: A Free Web Tool to Evaluate Pharmacokinetics, Drug-Likeness and Medicinal Chemistry Friendliness of Small Molecules. Sci. Rep..

[41] Sarkar S.M.A., Kumer A. (2021). Computational Investigation of Methyl α-d-Glucopyranoside Derivatives as Inhibitor against Bacteria, Fungi and COVID-19 (Sars-2). J. Chil. Chem. Soc..

[42] Punjabi M., Bharadvaja N., Sachdev A., Krishnan V. (2018). Molecular Characterization, Modeling, and Docking Analysis of Late Phytic Acid Biosynthesis Pathway Gene, Inositol Polyphosphate 6-/3-/5-Kinase, a Potential Candidate for Developing Low Phytate Crops. 3 Biotech.

[43] Wang J., Wolf R.M., Caldwell J.W., Kollman P.A., Case D.A. (2004). Development and Testing of a General Amber Force Field. J. Comput. Chem..

[44] Krieger Elmar G.V., Spronk C. (2013). YASARA–Yet Another Scientific Artificial Reality Application. YASARA Org.

[45] Harrach M.F., Drossel B. (2014). Structure and Dynamics of TIP3P, TIP4P, and TIP5P Water near Smooth and Atomistic Walls of Different Hydroaffinity. J. Chem. Phys..

[46] Krieger E., Vriend G. (2015). New Ways to Boost Molecular Dynamics Simulations. J. Comput. Chem..

[47] Harvey M.J., De Fabritiis G. (2009). An Implementation of the Smooth Particle Mesh Ewald Method on GPU Hardware. J. Chem. Theory Comput..

[48] Essmann U., Perera L., Berkowitz M.L., Darden T., Lee H., Pedersen L.G. (1995). A Smooth Particle Mesh Ewald Method. J. Chem. Phys..

[49] Krieger E., Nielsen J.E., Spronk C.A.E.M., Vriend G. (2006). Fast Empirical PKa Prediction by Ewald Summation. J. Mol. Graph. Model..

[50] Mahmud S., Rafi O., Paul G.K., Promi M.M., Shimu M.S.S., Biswas S., Bin Emran T., Dhama K., Alyami S.A., Moni M.A. (2021). Designing a Multi-Epitope Vaccine Candidate to Combat MERS-CoV by Employing an Immunoinformatics Approach. Sci. Rep..

[51] Mahmud S., Hasan M.R., Biswas S., Paul G.K., Afrose S., Mita M.A., Sultana Shimu M.S., Promi M.M., Hani U., Rahamathulla M. (2021). Screening of Potent Phytochemical Inhibitors Against SARS-CoV-2 Main Protease: An Integrative Computational Approach. Front. Bioinforma..

[52] Mahmud S., Paul G.K., Biswas S., Afrose S., Mita M.A., Hasan M.R., Shimu M.S.S., Hossain A., Promi M.M., Ema F.K. (2021). Prospective Role of Peptide-Based Antiviral Therapy Against the Main Protease of SARS-CoV-2. Front. Mol. Biosci..

[53] Mahmud S., Biswas S., Paul G.K., Mita M.A., Promi M.M., Afrose S., Hasan M.R., Zaman S., Uddin M.S., Dhama K. (2021). Plant-Based Phytochemical Screening by Targeting Main Protease of Sars-Cov-2 to Design Effective Potent Inhibitors. Biology.

[54] Kumar Paul G., Mahmud S., Aldahish A.A., Afroze M., Biswas S., Briti Ray Gupta S., Hasan Razu M., Zaman S., Salah Uddin M., Nahari M.H. (2022). Computational Screening and Biochemical Analysis of Pistacia Integerrima and Pandanus Odorifer Plants to Find Effective Inhibitors against Receptor-Binding Domain (RBD) of the Spike Protein of SARS-Cov-2. Arab. J. Chem..

[55] Mahmud S., Mita M.A., Biswas S., Paul G.K., Promi M.M., Afrose S., Hasan R., Shimu S.S., Zaman S., Uddin S. (2021). Molecular Docking and Dynamics Study to Explore Phytochemical Ligand Molecules against the Main Protease of SARS-CoV-2 from Extensive Phytochemical Datasets. Expert Rev. Clin. Pharmacol..

[56] Mahmud S., Biswas S., Kumar Paul G., Mita M.A., Afrose S., Robiul Hasan M., Sharmin Sultana Shimu M., Uddin M.A.R., Salah Uddin M., Zaman S. (2021). Antiviral Peptides against the Main Protease of SARS-CoV-2: A Molecular Docking and Dynamics Study. Arab. J. Chem..

[57] Jagannathan R. (2019). Characterization of Drug-like Chemical Space for Cytotoxic Marine Metabolites Using Multivariate Methods. ACS Omega.

[58] Defoirdt T., Boon N., Sorgeloos P., Verstraete W., Bossier P. (2007). Alternatives to Antibiotics to Control Bacterial Infections: Luminescent Vibriosis in Aquaculture as an Example. Trends Biotechnol..

[59] Ali F., Yao Z., Li W., Sun L., Lin W., Lin X. (2018). In-Silico Prediction and Modeling of the Quorum Sensing Luxs Protein and Inhibition of AI-2 Biosynthesis in Aeromonas Hydrophila. Molecules.

[60] Li W., Ali F., Cai Q., Yao Z., Sun L., Lin W., Lin X. (2018). Quantitative Proteomic Analysis Reveals That Chemotaxis Is Involved in Chlortetracycline Resistance of Aeromonas Hydrophila. J. Proteom..

[61] Bentzon-Tilia M., Sonnenschein E.C., Gram L. (2016). Monitoring and Managing Microbes in Aquaculture – Towards a Sustainable Industry. Microb. Biotechnol..

[62] Skjermo J., Vadstein O. (1999). Techniques for Microbial Control in the Intensive Rearing of Marine Larvae. Aquaculture.

[63] Orozco-Medina C., Maeda-Martínez A.M., López-Cortés A. (2002). Effect of Aerobic Gram-Positive Heterotrophic Bacteria Associated with Artemia Franciscana Cysts on the Survival and Development of Its Larvae. Aquaculture.

[64] Rajabi S., Ramazani A., Hamidi M., Naji T. (2015). Artemia Salina as a Model Organism in Toxicity Assessment of Nanoparticles. DARU, J. Pharm. Sci..

[65] Hamza F., Kumar A.R., Zinjarde S. (2018). Efficacy of Cell Free Supernatant from Bacillus Licheniformis in Protecting Artemia Salina against Vibrio Alginolyticus and Pseudomonas Gessardii. Microb. Pathog..

[66] Sofos J.N., Flick G., Nychas G.-J., O’Bryan C.A., Ricke S.C., Crandall P.G. (2014). Meat, Poultry, and Seafood. Food Microbiol..

[67] Hamza F., Kumar A.R., Zinjarde S. (2016). Antibiofilm Potential of a Tropical Marine Bacillus Licheniformis Isolate: Role in Disruption of Aquaculture Associated Biofilms. Aquac. Res..

[68] Padhi S., Masi M., Chourasia R., Rajashekar Y., Rai A.K., Evidente A. (2021). ADMET Profile and Virtual Screening of Plant and Microbial Natural Metabolites as SARS-CoV-2 S1 Glycoprotein Receptor Binding Domain and Main Protease Inhibitors. Eur. J. Pharmacol..

[69] Mahanta S., Chowdhury P., Gogoi N., Goswami N., Borah D., Kumar R., Chetia D., Borah P., Buragohain A.K., Gogoi B. (2020). Potential Anti-Viral Activity of Approved Repurposed Drug against Main Protease of SARS-CoV-2: An in Silico Based Approach. J. Biomol. Struct. Dyn..

[70] Parvathaneni V., Kulkarni N.S., Muth A., Gupta V. (2019). Drug Repurposing: A Promising Tool to Accelerate the Drug Discovery Process. Drug Discov. Today.

[71] Sayed A.M., Alhadrami H.A., El-Gendy A.O., Shamikh Y.I., Belbahri L., Hassan H.M., Abdelmohsen U.R., Rateb M.E. (2020). Microbial Natural Products as Potential Inhibitors of SARS-CoV-2 Main Protease (Mpro). Microorganisms.

