# Supplementary material for: Klebsiella pneumoniae Volatile Organic Compounds (VOCs) Protect Artemia salina from Fish Pathogen Aeromonas sp.: A Combined In Vitro, In Vivo, and In Silico Approach
Source: Microorganisms. 2023 Jan 10;11(1):172. doi: 10.3390/microorganisms11010172 (PMC9862385; doi:10.3390/microorganisms11010172)
Supplement: Supplementary file 1 [file microorganisms-11-00172-s001.zip › microorganisms-2039679-supplementary.pdf]

Klebsiella pneumoniae Volatile Organic Compounds (VOCs) Protect *Artemia salina* from Fish Pathogen *Aeromonas* sp.: A combined *In vitro, In vivo*, and *In silico* approach

Md. Liton Mahmud[1,#], Shirmin Islam[1,#], Suvro Biswas[1], Md. Golam Mortuza[2], Gobindo Kumar Paul[1,3], Md. Salah Uddin[1], Md. Akhtar-E-Ekram[1], Md. Abu Saleh[1,*] and Shahriar Zaman[1,*], Asad Syed[4], Abdallah M.Elgorban[4], Nouf S. S. Zaghloul[5]

[1]Microbiology Laboratory, Department of Genetic Engineering and Biotechnology, University of Rajshahi, Rajshahi-6205, Bangladesh
[2]Department of Science and Humanities, Bangladesh Army International University of Science and Technology, Cumilla-3500, Bangladesh
[3]Bangladesh Reference Institute for Chemical Measurements (BRiCM), Bangladesh Council of Scientific and Industrial Research (BCSIR), Dhaka, Bangladesh
[4]Department of Botany and Microbiology, College of Science, King Saud University, P.O. 2455, Riyadh, 11451, Saudi Arabia
[5]Bristol Centre for Functional Nanomaterials, HH Wills Physics Laboratory, Tyndall Avenue, University of Bristol, Bristol, BS8 1FD, UK.

# Indicates equal contribution
* Correspondence: to: saleh@ru.ac.bd ; szaman@ru.ac.bd

Table S1: List of bacterial strains used for antagonistic test.

Sl. no.	Name of the used bacterial strains
**01**	*Staphylococcus aureus*
**02**	*Lactobacillus* sp.
**03**	*Bacillus thuringiensis*
**04**	*Klebsiella pneumoniae*
**05**	*Pseudomonas* sp.
**06**	*Escherichia coli*

Table S2: Volatile compounds (with PubChem ID, molecular weight and binding affinity) produced by *Klebsiella Pneumoniae*. Data were obtained from GC-MS analysis

	Compound Names	PubChem ID	Molecular Weight (g/mol)	Binding Affinity
**L-1**	Butanoic acid, methyl ester	12180	102.13	-4.5
**L-2**	1-Butanol, 3-methyl-	31260	88.15	-4.5
**L-3**	Disulfide, dimethyl	12232	94.2	-2.4
**L-4**	2,2-Dichloroethanol, acetate	527751	156.99	-4.6
**L-5**	Butanoic acid	264	88.11	-4.6
**L-6**	Butanoic acid, 3-methyl-	10430	102.13	-4.4
**L-7**	Butanoic acid, 2-methyl-	8314	102.13	-4.9
**L-8**	Oxime-, methoxy-phenyl-_	9602988	151.16	-6.7
**L-9**	2-Hydroxymethyl-6-methoxytetrahydropyran-	360938	162.18	-4.9

	3-ol			
**L-10**	Oxime-, methoxy-phenyl-_	9602988	151.16	-6.7
**L-11**	Disulfide, fluoromethyl methyl	574430	112.19	-2.7
**L-12**	Anisole	7519	108.14	-5.3
**L-13**	4',6'-Dimethoxy-2',3'-dimethylacetophenone	606430	208.25	-5.3
**L-14**	Butanoic acid, 2-methylpropyl ester	10885	144.21	-4.8
**L-15**	Dimethyl trisulfide	19310	126.3	-2.3
**L-16**	Pentasulfide, dimethyl	81772	190.4	-2.4
**L-17**	2,4-Dichloro-6-[(3-hydroxyphenylimino)methyl]phenol	548611	282.12	-6.1
**L-18**	Phenol, 2-chloro-	7245	128.55	-5.7
**L-19**	Butanoic acid, butyl ester	7983	144.21	-4.2
**L-20**	Pyrazine, trimethyl-	26808	122.17	-5.1
**L-21**	Propanoic acid, 2-methyl-, 3-methylbutyl ester	519786	158.24	-4.2
**L-22**	(Z)-3,7-Dimethyl-2,7-octadien-1-ol, propanoate(ester)	5365067	210.31	-4.6
**L-23**	2-Ethyl-1-hexanol, pentafluoropropionate	545245	276.24	-5.3
**L-24**	Butanoic acid, 3-methyl-, butyl ester	7981	158.24	-5.0
**L-25**	Butyl 2-methylbutanoate	61812	158.24	-4.2
**L-26**	Propanoic acid, 2-methyl-, 3-methylbutyl ester	519786	158.24	-4.2
**L-27**	Propanoic acid, 2-methyl-, pentyl ester	75554	158.24	-4.1
**L-28**	Phenol, 2-methyl-	335	108.14	-5.9
**L-29**	2,5-Dihydroxybenzaldehyde, 2TMS derivative	622536	282.48	-2.6
**L-30**	Pentanoic acid, pentyl ester	62433	172.26	-5.3
**L-31**	Phenylethyl Alcohol	6054	122.16	-6.0
**L-32**	Fluoren-9-ol, 3,6-dimethoxy-9-(2-phenylethynyl)-	631096	342.4	-7.1
**L-33**	Pyrazine, 3,5-diethyl-2-methyl-	28906	150.22	-5.2

**L-34**	1-Methoxy-2-methyl-4-(methylthio)benzene	592820	168.26	-4.3
**L-35**	Octanoic acid	379	144.21	-5.0
**L-36**	Neophytadiene	10446	278.5	-4.2
**L-37**	Naphthalene	931	128.169	-6.2
**L-38**	Octane, 2-bromo-	79046	193.12	-4.2
**L-39**	Pentasulfide, dimethyl	81772	190.4	-2.4
**L-40**	Benzothiazole	7222	135.19	-4.6
**L-41**	Methylcyclohexylacetate	139743	156.22	-6.3
**L-42**	16-Methyl-heptadecane-1,2-diol, trimethylsilyl ether	91742675	430.9	-2.7
**L-43**	Nonanoic acid	8158	158.24	-4.3
**L-44**	Docosanoic acid, docosyl ester	87221	649.2	-3.5
**L-45**	16-Methyl-heptadecane-1,2-diol, trimethylsilyl ether	91742675	430.9	-2.5
**L-46**	2e)-2-(Hydroxyimino)-3-Phenylpropanoic Acid-	6399465	179.17	-5.4
**L-47**	2,4,5,6,8-Pentathianonane	10420954	218.5	-2.7
**L-48**	Cetene	12395	224.42	-3.7
**L-49**	Tetradecane	12389	198.39	-4.1
**L-50**	Dodecanal	8194	184.32	-3.9
**L-51**	3,9-Dimethyl-4,8-diaza-3,8-undecadiene-2,10-dione dioxime	135472033	240.3	-5.8
**L-52**	2,6,10,14-Tetramethyl-7-(3-methylpent-4-enylidene) pentadecane	91694785	348.6	-4.5
**L-53**	Benzeneacetic acid, 2-methylpropyl ester	60998	192.25	-5.4
**L-54**	Hexanoic acid, 2-phenylethyl ester	61384	220.31	-5.1
**L-55**	2,5-Cyclohexadiene-1,4-dione, 2,6-bis(1,1-dimethylethyl)-	12867	220.31	-5.2
**L-56**	1-Tetradecene	14260	196.37	-4.1

**L-57**	Dimethyl trisulfide	19310	126.3	-2.3
**L-58**	2H-Indol-2-one, 1,3-dihydro-	321710	133.15	-6.4
**L-59**	Pentadecane	12391	212.41	-4.0
**L-60**	Phenol, 3,5-bis(1,1-dimethylethyl)-	70825	206.32	-5.6
**L-61**	N1,N1,N4-Tris(tert-butyldimethylsilyl)succinamide	91744783	458.9	-3.0
**L-62**	Hexathiane	139602	192.4	-2.3
**L-63**	Octadecane, 1-bromo-	8218	333.4	-3.8
**L-64**	1,3,3-Trimethyl-2-(2-methyl-cyclopropyl)-cyclohexene	595941	178.31	-5.5
**L-65**	Heptadecane	12398	240.5	-3.9
**L-66**	Decanoic acid, decyl ester	74247	312.5	-4.9
**L-67**	1,4-Methanobenzocyclodecene, 1,2,3,4,4a,5,8,9,12,12a-decahydro-	556414	202.33	-5.6
**L-68**	Oxalic acid, cyclohexylmethyl tridecyl ester	6421725	368.5	-5.0
**L-69**	ethanol, 2-(dodecylsulfinyl)-	88528	262.45	-4.4
**L-70**	2,4-Di-tert-butylthiophenol	519681	222.39	-5.1
**L-71**	Ethanol, 2-(tetradecyloxy)-	16491	258.44	-4.4
**L-72**	Hexathiepane	87012	206.4	-2.6
**L-73**	2-Dodecen-1-yl(-)succinic anhydride	5362708	266.38	-4.8
**L-74**	3,5-di-tert-Butyl-4-hydroxyacetophenone	616296	248.36	-5.5
**L-75**	2,6-Bis(1,1-dimethylethyl)-4-(1-oxopropyl)phenol	616172	262.4	-5.2
**L-76**	7,9-Di-tert-butyl-1-oxaspiro(4,5)deca-6,9-diene-2,8-dione	545303	276.4	-5.9
**L-77**	Dibutyl phthalate	3026	278.34	-5.8

Table S3: Protein-ligand interactions of top eight hit molecules with their binding residues.

L/N	Volatile Compounds	2D Diagram (PDB ID: 5b7n)	Hydrogen bond Amino Acids Residues	Hydrophobic Amino Acid Residues
L-32		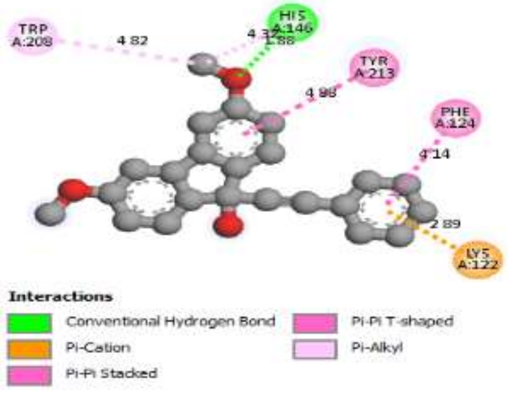	HIS-146(1.88)	TRP-208(4.82) TYR-213(4.88) PHE-124(4.14) LYS-122(2.89)
L-8		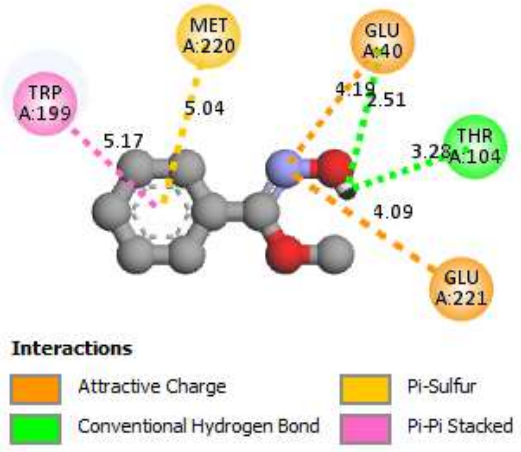	THR-104(3.28)	MET-220(5.04) GLU-40(4.19) GLU-221(4.09) TRP-199(5.17)

L-58		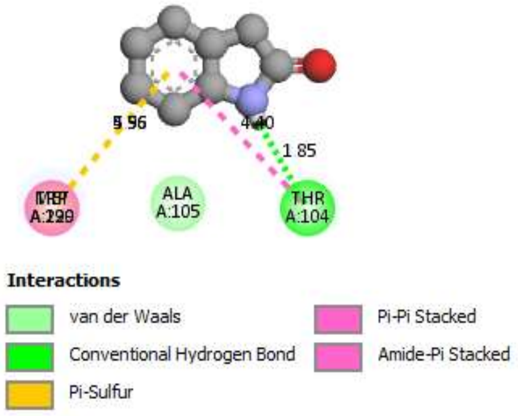	THR-104(1.85)	MET-220(5.96)
L-41		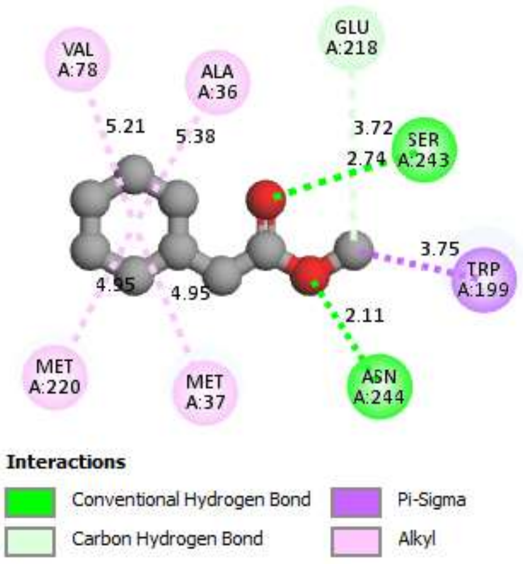	SER-243(2.74) ASN-244(2.11)	VAL-78(5.21) ALA-36(5.38) GLU-218(3.72) TRP-199(3.75) MET-37(4.95) MET-27(4.95) MET-220 (4.95)

L-37		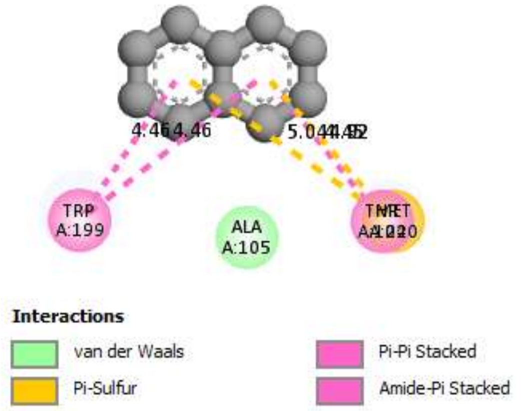	Absent	TRP-199 (4.45)
L-17		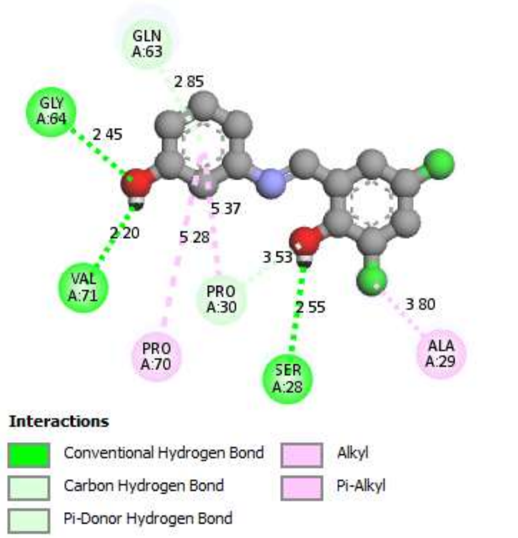	SER-28(2.55) VAL-71(2.20) GLY-64(2.45)	GLN-63(2.85) PRO-70(5.28) PRO-30(5.37) PRO-30(5.37) ALA-29(3.80)
L-31		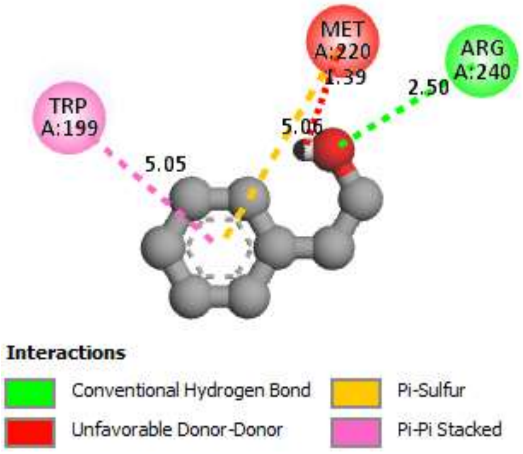	ARG-240(2.50)	MET-220(1.39) MET-220(5.05) TRP-199(5.05) ARG-240(2.50)

L-76		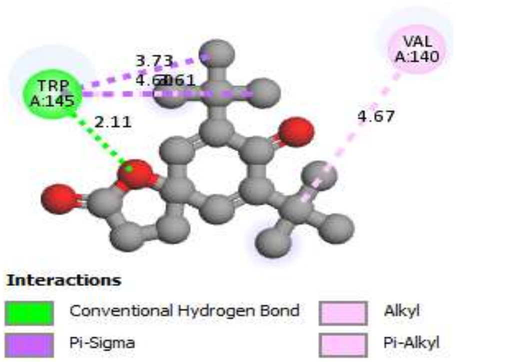	TRP-145(2.11)	TRP-145 (3.73)<br>TRP-145(3.73)<br>TRP-145(4.60)<br>VAL-140(4.67)

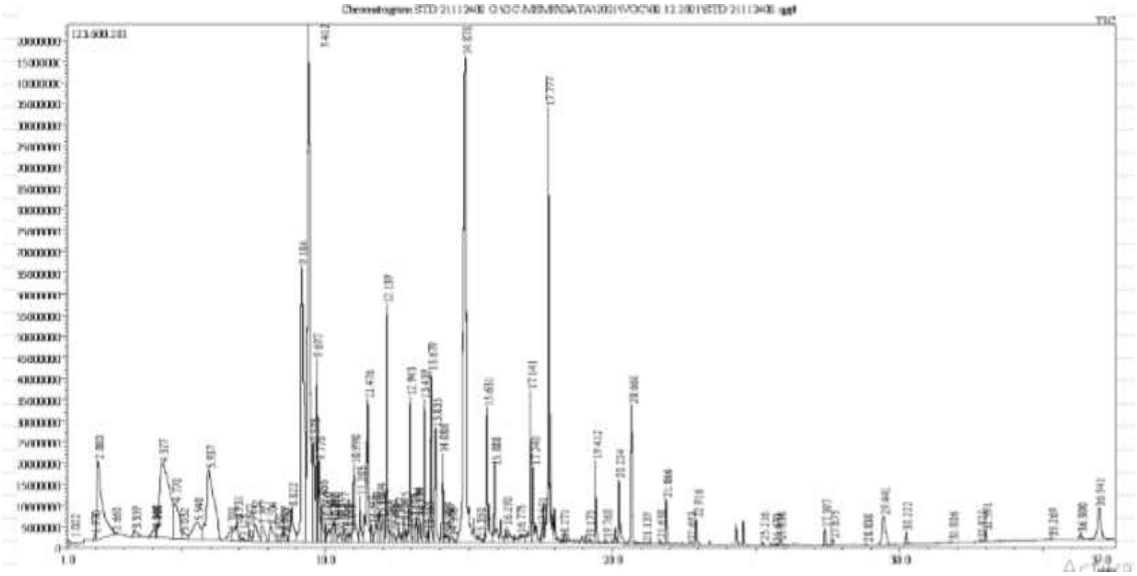

Figure S1: GC-MS chromatogram of volatile compounds produced by *Klebsiella pneumoniae*.
